# Degradation and selective ligninolysis of wheat straw and banana stem for an efficient bioethanol production using fungal and chemical pretreatment

**DOI:** 10.1007/s13205-012-0102-4

**Published:** 2012-11-15

**Authors:** Shilpi Thakur, Bhuvnesh Shrivastava, Snehal Ingale, Ramesh C. Kuhad, Akshaya Gupte

**Affiliations:** 1 Department of Microbiology, Natubhai V. Patel College of Pure and Applied Sciences, Vallabh Vidyanagar, 388120 Gujarat India; 2 Lignocellulose Biotechnology Laboratory, Department of Microbiology, University of Delhi, South Campus, New Delhi, 110021 India; 3 Ashok and Rita Patel Institute of Integrated Study and Research in biotechnology and Allied Sciences, New Vallabh Vidyanagar, 388121 Gujarat India

**Keywords:** Lignocelluloses, Pretreatment, *Pleurotus ostreatus* HP-1, Bioethanol, Saccharification

## Abstract

Lignocelluloses from agricultural, industrial, and forest residues constitute a majority of the total biomass present in the world. Environmental concerns of disposal, costly pretreatment options prior to disposal, and increased need to save valuable resources have led to the development of value-added alternate technologies such as bioethanol production from lignocellulosic wastes. In the present study, biologically pretreated (with the fungus, *Pleurotus ostreatus* HP-1) and chemically pretreated (with mild acid or dilute alkali) wheat straw (WS) and banana stem (BS) were subsequently subjected to enzymatic saccharification (with mixture of 6.0 U/g of filter paper cellulase and 17 U/g of β-glucosidase) and were evaluated for bioethanol production using *Saccharomyces cerevisiae* NCIM 3570. Biological and chemical pretreatments removed up to 4.0–49.2 % lignin from the WS and BS which was comparatively higher than that for cellulose (0.3–12.4 %) and for hemicellulose (0.7–21.8 %) removal with an average 5.6–49.5 % dry matter loss. Enzymatic hydrolysis yielded 64–306.6 mg/g (1.5–15 g/L) reducing sugars from which 0.15–0.54 g/g ethanol was produced from *Saccharomyces cerevisiae* NCIM 3570.

## Introduction

Industrial bioconversion of renewable resources to bioethanol is a promising alternative to petroleum-based chemical synthesis (Volynets and Dahman 2011). In this context, lignocelluloses, the most abundant source of sugars, are a potential candidate for obtaining energy in the form of charcoal, hydrogen, ethanol, and biogas; the last three of which require hydrolysis of the lignocellulosic material. Production of biofuel and other bioproducts from lignocellulose is expected to promote rural economies, enhance energy security, and alleviate environmental pollution (Espino et al. 2011).

The use of lignocelluloses as a raw material is particularly convenient because it results in a significant reduction in the cost of production of value-added products viz. enzymes, organic acids, etc. However, for the establishment of lignocellulose-based biofuel economy, saccharification of these materials is necessary (Espino et al. 2011). The phenyl propanoid polymers in the lignin prevents access to the cellulose and hemicellulose polymer for the efficient release of sugars, and subsequent fermentation to bioethanol, and hence the lignin must be removed first (Espino et al. 2011). During the last decade, considerable developments in pretreatment techniques for improving the rate of release and total yield of sugars in the hydrolysis step have been made (Hendriks and Zeeman 2009) and using these processes substantial amounts of lignin and hemicelluloses have been hydrolyzed and transformed into value-added products (Dashtban et al. 2009). The pretreatment techniques can be classified as physical, chemical, and biological and can be used individually or in combination, such as mechanical and thermal or chemical and thermal (Talebnia et al. 2010). The overall efficiency of any pretreatment process can be correlated to a good balance between low inhibitor formation and high substrate digestibility.

Biological pretreatment comprises the use of microorganisms especially brown rot, soft rot and/or white-rot fungi to selectively degrade lignin and hemicellulose. Of these, white-rot fungi seem to be the most effective as they are potential producers of laccase (E.C.1.10.3.2), manganese peroxidase (MnP; E.C.1.11.1.13), and lignin peroxidase (LiP; E.C.1.11.1.14), enzymes that modify lignin. Various researchers have reported the use of many white-rot fungi (*Pleurotus ostreatus*, *Trametes versicolor*, *Phanerochaete chrysosporium*, and *Pycnoporus cinnabarinus*) for the depolymerization of lignin and hemicellulosic compounds (Kuhar et al. 2008; Shrivastava et al. 2011). The selection of fungi is based on their higher affinity to degrade lignin rather than the carbohydrate moieties. Though the use of biological pretreatment is considered safe, environmentally friendly and less energy consuming compared to other pretreatment methods, the rate of hydrolysis is low and needs improvement to make it a commercially viable proposition.

The objective of the present work was to evaluate and compare the effect of fungal pretreatment with the chemical pretreatments on Wheat straw (WS) and banana stem (BS) and to improve the enzymatic hydrolysis to obtain higher reducing sugar for bioethanol production.

## Materials and methods

### Lignocellulosic substrates

Wheat straw (WS) and banana stem (BS) were collected locally and ground to an approximate particle size of 1–2 mm. For analytical purposes, the untreated and pretreated substrates were milled to 30 mesh size.

### Microorganisms and culture conditions

*Pleurotus ostreatus* HP-1 (GenBank accession no. EU420068) was grown and maintained on 2 % Malt Extract Agar (MEA) medium agar plates. MEA contained (g/L) Malt extract 30.0, Peptone 3.0, and Agar 15.0 (pH 5.0) at 30 °C. *Saccharomyces cerevisiae* NCIM 3570 was procured from National Collection of Industrial Microorganisms (NCIM), National Chemical Laboratory (NCL), Pune, India and was maintained on agar slants containing (g/L) Peptone 10.0, Glucose 40.0, NaCl 5.0, and agar 20.0 (pH 5.5) at 30 °C. Both isolates were stored at 4 °C and sub-cultured every fortnight.

The inoculum of *Pleurotus ostreatus* HP-1 was prepared in 50 ml 2 % Malt Extract Broth (MEB) in 250 ml Erlenmeyer flasks. MEB had the same composition as MEA but lacked agar. The flasks were inoculated with 5 mycelial discs (7 mm diameter each) which had been prepared from 8-day-old fungal culture grown on MEA and incubated on a rotatory shaker (150 rpm) at 30 °C for 8 days. The inoculum of *S. cerevisiae* was prepared by growing the culture at 30 °C for 24 h under static condition in a medium containing (g/L) Peptone 10.0, Glucose 40.0, and NaCl 5.0 (pH 5.5).

### Pretreatment of substrates

#### Chemical pretreatment

WS and BS were subjected to two chemical pretreatment regimes prior to enzymatic hydrolysis and fermentation. For this, a 10 % slurry of WS or BS was incubated in 1 N NaOH or 1 N H_2_SO_4_ at room temperature for 24 h following which the slurries were washed repeatedly with water to a neutral pH, and then oven dried at 60 °C to a constant weight.

#### Fungal (biological) pretreatment

Fungal pretreatment was carried out with 15.0 g of WS or BS in 500 ml Erlenmeyer flasks and were moistened with a medium described by Asther et al. (1988) to give a final substrate to moisture ratio of 1:4 (w/v). The moistening medium containing (g/L) KH_2_PO_4_ 0.2, CaCl_2_·2H_2_O 0.0132, MgSO_4_·7H_2_O 0.05, FeC_6_H_5_O_7_·NH_4_OH 0.085, ZnSO_4_·7H_2_O 0.0462, MnSO_4_·7H_2_O 0.035, CoCl_2_·6H_2_O 0.007, CuSO_4_·5H_2_O 0.007, l-Aspargine 1.0, NH_4_NO_3_ 0.5, Thiamine-HCl 0.0025, yeast extract 0.5, Glucose 10, Tween-80 0.1, and pH 5.0. Each flask was inoculated with *P. ostreatus* HP-1 to a ratio of 65 mg fungal dry mass per gram of BS or WS and incubated at 30 °C for various incubation periods. The flasks without fungal biomass served as a control.

### Enzyme assays

The cultures in the flasks harvested at intervals of 4 days, suspended in 30 mL acetate buffer (pH 5.0, 100 mM) and shaken in a rotatory shaker at 150 rpm at 30 °C. After 2 h incubation, the cultures were filtered through muslin cloth, and the cloth squeezed to maximize enzyme extraction. The filtrate was centrifuged at 8,000 rpm at 4 °C for 15 min and the supernatant was analyzed for laccase, peroxidase, xylanase, and β-Glucosidase activities. Laccase activity (E.C. 1.10.3.2) was determined by monitoring the A_420_ change related to the rate of oxidation of 2, 2-Azino- Bis-3-ethyl-benzthiozoline-6-sulphonic acid (ABTS, ε = 36,000 cm^−1^ M^−1^) at 30 °C for 3 min (Niku-Paavola et al. 1990). The reaction mixture contained 100 μL of 50 mM ABTS, 800 μL of 100 mM Na-Acetate buffer (pH 5.0), and 100 μL of appropriately diluted enzyme extract. Manganese peroxidase (MnP) activity (E.C. 1.11.1.13) was measured by oxidation of 2, 6-dimethoxy phenol (DMP, ε = 27,500 cm^−1^ M^−1^) in the presence of H_2_O_2_ and MnSO_4_ at 469 nm. The reaction mixture contained 1 mM DMP, 0.1 mM H_2_O_2_, 1 mM MnSO_4_, and 100 mM Sodium tartarate buffer (pH 4.5). MnP activity was corrected for manganese-independent peroxidase activity by subtracting the activity obtained at pH 3.25 in the absence of MnSO_4_ at 469 nm (Martinez et al. 1996). One unit of enzyme activity (U) was defined as the amount of enzyme which leads to the oxidation of 1 μM of substrate/min under the standard assay condition. Xylanase (EC 3.2.1.8) activity was determined according to Bailey et al. (1992) using birch wood xylan (1 % w/v) as a substrate. Filter paper activity (FPase) was determined according to Ghose (1987) using Whatman no. 1 filter paper as a substrate. One unit of enzyme activity is defined as the amount of enzyme that liberates 1 μmole of reducing sugar as xylose or glucose per min under the standard assay condition. *β*-glucosidase activity was determined by measuring *p*-nitrophenol released from *p*-nitrophenyl *β*-D-glucopyranoside at 50 °C. The reaction mixture consisting of 2 mM *p*-nitrophenyl *β*-D-glucopyranoside in 50 mM sodium citrate buffer (pH 4.8) was incubated with enzyme at 50 °C for 30 min in a total volume of 0.5 mL. The reaction was terminated by addition of 1 mL 2 M sodium carbonate. The amount of *p*-nitrophenol released was determined by measuring absorbance at 410 nm. One unit of β-Glucosidase activity is defined as amount of enzyme required to release 1 μM *p*-nitrophenol per minute under the standard assay condition.

### Enzymatic hydrolysis

Enzymatic hydrolysis of untreated and pretreated WS and BS was performed using in-house produced crude cellulase from mixed culture of *Aspergillus fumigatus* and *Aspergillusellipticus.* The reaction system for saccharification consisted of 100 ml of sodium citrate buffer (0.05 M, pH 5.0) containing 5 g of respective substrate (untreated and pretreated) and a mixture of 6.0 U/g of crude filter paper cellulase (FPase) and 17 U/g of β-glucosidase. The reaction was carried out at 50 °C and 150 rpm for 48 h. The samples were withdrawn at regular interval of 4 h, centrifuged at 8,000 rpm for 15 min and the supernatant was analyzed for total reducing sugar released by dinitrosalicylic acid method (Miller 1959).

### Ethanol production

The enzymatic hydrolysates obtained were supplemented with (g/L) (NH_4_)_2_SO_4_ 0.5, KH_2_PO_4_ 0.5, yeast extract 2.5, pH 5.5, and inoculated with 2.0 % (v/v) culture of *S. cerevisiae* NCIM 3570 (O.D. 0.6) and incubated at 30 °C for 48 h. The fermented broth obtained was centrifuged at 10,000 rpm at 4 °C for 10 min and the cell-free supernatant was used for the analysis of ethanol.

### Analytical methods

Weight loss of substrate was determined by subtracting the weight of oven dried pretreated substrate at 60 °C to a constant weight from the weight of control (untreated) substrate. The content of cellulose, hemicellulose and lignin were determined by methods described by Van Soest (Goering and Van Soest 1970; Van Soest et al. 1991). Total reducing sugars were estimated by the dinitrosalicylic acid (DNSA) method (Miller 1959).

Ethanol was estimated by gas chromatography (GC) (Perklin Elmer, Turbometrix 40 headspace sampler) with PE wax column at oven temperature of 85 °C and flame ionization detector (FID) at 200 °C. Nitrogen was used as a carrier gas with a flow rate of 0.5 mL/min. The ethanol standards were prepared using commercial grade ethanol (Merck, India).

All the experiments were carried out in triplicates and the data represent the mean values of the experiments.

## Results and discussion

### Chemical pretreatment

Chemical Pretreatment employed different chemicals such as acid and alkali. The purpose of pretreatment of substrates is to disorganize the crystalline structure of micro and macro fibrils to release polymer chains of cellulose and hemicelluloses and modify the pore size to render them accessible to enzymatic attack. Among the various pretreatments, dilute acid and mild alkali pretreatments are effective in targeting hemicelluloses and lignin, respectively (Talebnia et al. 2010). Table 1a depicts the chemical composition of untreated (control) WS and BS. In the present study, mild alkali (1 N NaOH) and dilute acid (1 N H_2_SO_4_) pretreatments were evaluated on each lignocellulosic substrate (WS and BS) separately. Table 1b summarizes the compositional changes during chemical pretreatment of WS and BS. Alkali pretreatment caused a substantial higher removal of lignin, cellulose, and dry matter of 46.3, 12.4, and 42 %, respectively, compared to acid pretreatment, i.e., 13.7, 7.2 and 24.9 %, respectively, in WS. However, the removal of hemicelluloses (21.8 %) was more with acid pretreatment as compared with alkali pretreatment (18.8 %). Similar trend of results were also obtained in case of BS with 49.2, 8.5 and 49.5 % of removal of lignin, cellulose and dry matter, respectively, in alkali pretreatment, while 16.2 % removal of hemicellulose was attained using acid pretreatment. According to the previous report (Talebnia et al. 2010), acid pretreatment helped in removing the hemicellulosic fraction and may have decreased the degree of polymerization during pretreatment. Dilute acid treatment is already reported not to remove the lignin completely from the substrate but modify the lignin–carbohydrate linkage (Dashtban et al. 2009). Moreover, the purpose of mild alkali pretreatment is to remove lignin and hence to increase the susceptibility of cellulose to become readily available for enzymes, which permits the yeast to convert the glucose into ethanol. The results thus obtained in the present study are in agreement with Bjerre et al. (1996) who have also reported dilute NaOH pretreatment as an effective method for lignin removal. Similarly in another study, Damisa et al. (2008) reported, pretreatment of substrates with alkali may result in the swelling of the particle causing easy lignin removal and cellulose depolymerization.

**Table 1:** (a) Chemical composition of (g/100 g dry matter) untreated (control) WS and BS and (b) Compositional changes during the chemical pretreatment of WS and BS

Components	Substrates			
	Wheat straw	Banana stem		
(a) Chemical composition of (g/100 g dry matter) untreated (control) WS and BS				
Cellulose	41.7 ± 1.10	40.8 ± 1.53		
Hemicellulose	28.05 ± 0.58	29.9 ± 1.14		
Lignin	7.9 ± 0.21	6.57 ± 0.78		

Components	Substrates			
	Wheat straw		Banana stem	
	Alkali pretreated	Acid pretreated	Alkali pretreated	Acid pretreated
(b) Compositional changes during the chemical pretreatment of WS and BS				
Dry matter loss (%)	42.0 ± 1.41	24.9 ± 0.63	49.5 ± 0.35	25.0 ± 0.17
Cellulose loss (%)	12.4 ± 0.38	7.2 ± 0.17	8.5 ± 0.42	6.8 ± 0.10
Hemicellulose loss (%)	18.8 ± 0.49	21.8 ± 0.56	15.3 ± 0.21	16.2 ± 0.49
Lignin loss (%)	46.3 ± 1.62	13.7 ± 0.32	49.2 ± 0.13	31.4 ± 0.28

### Fungal pretreatment

Biological pretreatment uses microorganisms and their enzymatic machinery for selective delignification of the lignocellulosic residues (Adenipekun and Fasidi 2005). The one of the main purpose of the biological pretreatment using white-rot basidiomycetes is to make substrate-carbohydrate digestible as much as possible and reduce environmental hazard (Adenipekun and Fasidi 2005). In the present study, white-rot basidiomycete *Pleurotus ostreatus* HP-1 was used for fermentation of WS and BS which resulted in decrease in lignin, cellulose, hemicellulose, and dry matter content (Table 2). Higher dry matter loss (DML) in WS (40 %) was obtained as compared with BS (32 %) after 32 days of fermentation which may be attributed to the collective removal of lignin, cellulose and hemicellulose (Gupta et al. 2011). Removal of lignin, cellulose, and hemicellulose occurred at a slow pace during whole fermentation period. Lignin removal was higher in WS (40 %) compared to BS (29 %) with a decrease in cellulose (10.4 and 7.3 %, respectively) and hemicellulose (12.9 and 14.3 %, respectively) content after 32 days of fermentation. This could be because of difference in cell wall assembly and chemical structure of lignin and lignin–carbohydrate complex present in WS and BS (Shrivastava et al. 2011). Lignin removal over the entire fermentation period obtained in the present study is found to be in the range of previously reported values of lignin degradation (up to 46 %) of plant residue by much studied *Phanerochaete flavido*-*alba* (Lopez et al. 2006). An increase in dry matter loss along with lignin, cellulose, and hemicellulose removal has also been reported by various authors (Shrivastava et al. 2011; Arora and Sharma 2009). In similar context, Hatakka et al. (2001) reported lignin degradation by *Lentinus edodes* occurred during secondary metabolism and nitrogen starvation condition which might have been the probable reason for achieving higher lignocellulosic degradation in the later phase of the present experiment. Various researchers have also reported similar degradative capability of *P. ostreatus* and have shown dry matter loss, hemicellulose, cellulose, and lignin removal ranging from 5 to 20 %, 2–20 %, 5–30 % and 2–40 % respectively (Shrivastava et al. 2011; Shabtay et al. 2009; Binod et al. 2010). There are several other reports where *Phanerochaete chrysosporium,* the most widely studied white-rot fungus for lignin degradation resulted in higher amount of lignin loss but was also found to cause significant loss in carbohydrate fraction (Shrivastava et al. 2011; Taniguchi et al. 2005).

**Table 2:** Compositional changes during the fungal pretreatment of WS and BS

Time (Days)	Substrates							
	Wheat straw				Banana stem			
	Dry matter loss (%)	Cellulose loss (%)	Hemicellulose loss (%)	Lignin loss (%)	Dry matter loss (%)	Cellulose loss (%)	Hemicellulose loss (%)	Lignin loss (%)
4	7.0 ± 0.21	2.0 ± 0.05	1.9 ± 0.10	4.0 ± 0.07	5.6 ± 0.17	0.3 ± 0.02	0.7 ± 0.10	6.4 ± 0.15
8	17.0 ± 0.63	2.6 ± 0.04	3.8 ± 0.14	7.5 ± 0.10	10.9 ± 0.50	0.5 ± 0.02	2.8 ± 0.05	10.4 ± 0.10
12	22.0 ± 0.56	3.0 ± 0.05	5.7 ± 0.09	20.0 ± 0.35	16.4 ± 0.60	3.5 ± 0.05	6.9 ± 0.15	12.9 ± 0.17
16	25.0 ± 0.17	4.3 ± 0.05	6.3 ± 0.12	21.5 ± 0.14	21.0 ± 0.42	3.9 ± 0.05	9.4 ± 0.20	14 ± 0.12
20	28.0 ± 0.13	4.4 ± 0.05	7.4 ± 0.15	26.5 ± 1.0	26.6 ± 0.66	4.5 ± 0.04	9.9 ± 0.10	16.1 ± 0.20
24	30.0 ± 1.00	5.7 ± 0.10	9.7 ± 0.05	29.7 ± 0.49	29.5 ± 0.90	5.4 ± 0.03	10.6 ± 0.15	20.9 ± 0.20
28	34.0 ± 0.91	8.9 ± 0.10	11.8 ± 0.20	39.0 ± 0.70	30.7 ± 1.20	5.9 ± 0.10	13.6 ± 0.15	24.0 ± 0.59
32	40.0 ± 0.70	10.4 ± 0.30	12.9 ± 0.18	40.0 ± 0.70	32.0 ± 1.15	7.3 ± 0.15	14.3 ± 0.30	29.0 ± 1.20

**Fig. 1:**
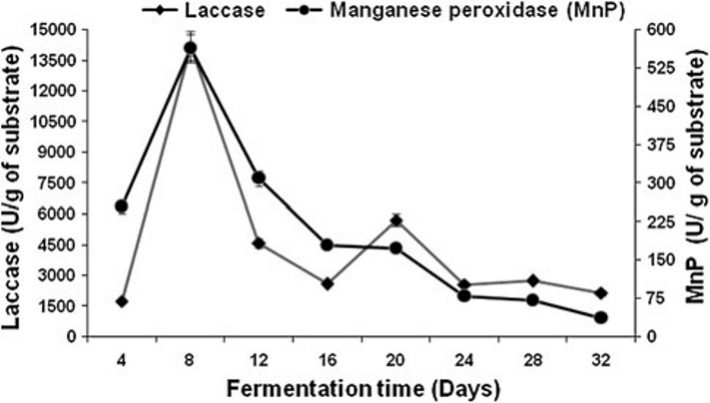
Production pattern of ligninolytic enzymes on WS by *P. ostreatus* HP-1

**Fig. 2:**
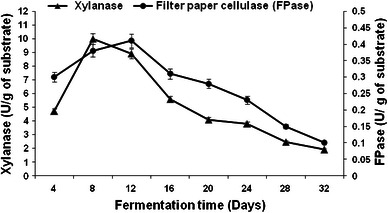
Production pattern of hydrolytic enzymes on WS by *P. ostreatus* HP-1

**Fig. 3:**
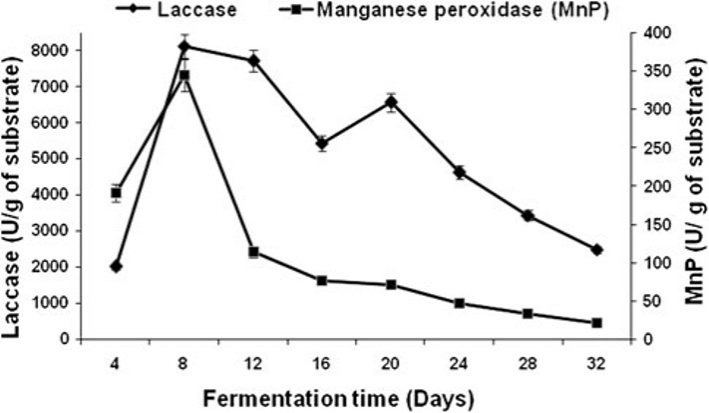
Production pattern of ligninolytic enzymes on BS by *P. ostreatus* HP-1

**Fig. 4:**
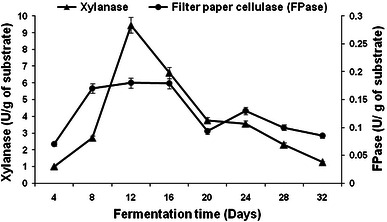
Production pattern of hydrolytic enzymes on BS by *P. ostreatus* HP-1

The difference in the degradation pattern of substrate by *Pleurotus ostreatus* HP-1 could be attributed to the different enzymatic profile observed during growth on WS and BS (Figs. 1, 2, 3, 4). Maximum laccase activity of 14,189 and 8,118.5 U/g, and 562.80 and 344.57 U/g of manganese peroxidase were obtained on 8th day of fermentation with WS and BS, respectively (Figs. 1, 3). Thereafter, no significant increase in the MnP production was observed during entire fermentation period. Initially, in the lag phase the activity increases slowly reaching maximum on the 8th day for laccase but thereafter decreases. In the late phase of cultivation, increase in laccase activity was again observed. The twin peak in laccase production could be attributed to the mycelial autolysis and release of mycelial bound enzyme or it might be due to nitrogen starvation in the second phase of cultivation (Shrivastava et al. 2011; Gupte et al. 2007). Similar pattern of laccase activity in two *Pleurotus* spp has also been reported by Reddy et al. (2003). Xylanase activities of 10 and 9.45 U/g of substrate were obtained on 8th day of fermentation with WS and BS, respectively (Figs. 2, 4).

The degradation of WS and BS (in terms of dry matter, lignin, cellulose, and hemicellulose removal) continued throughout the studied period and substrate degradation was not found to be directly correlated with the production of enzymes (Table 2, Figs. 1, 2) as has been reported by Li et al. (2008) and Mendonca et al. (2008). Recently, Sharma and Arora (2010) and Shrivastava et al. (2011) have also observed that the enzyme production profile cannot be correlated well with the degradation of polymer, which showed that fiber degradation not only depends upon the production of enzymes but also regulated by a variety of physiological factors.

### Enzymatic hydrolysis

**Fig. 5:**
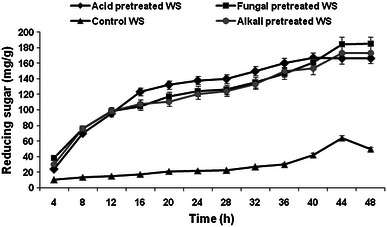
Enzymatic hydrolysis profile of untreated and pretreated WS

**Fig. 6:**
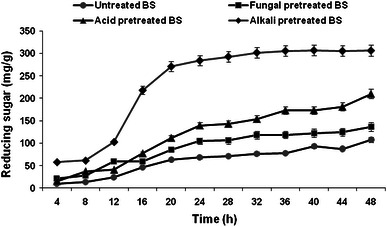
Enzymatic hydrolysis profile of untreated and pretreated BS

Pretreatment is a crucial step for any lignocelluloses prior to enzymatic hydrolysis (saccharification). Different pretreatments have been reported in the literature to make substrate more conducive for hydrolysis (Zhao et al. 2010). To evaluate the effect of pretreatment conditions on the digestibility of WS and BS, enzymatic hydrolysis was carried out and results are depicted in Figs. 5 and 6. Maximum reducing sugar of 64 mg/g (3.2 g/L), 184.8 mg/g (9.23 g/L), 173 mg/g (8.65 g/L), and 166 mg/g (8.35 g/L) were obtained with untreated, fungal pretreated, alkali pretreated, and acid pretreated WS, respectively, in 44 h. Whereas, in case of BS it was 107.8 mg/g (5.3 g/L), 136.8 mg/g (6.8 g/L), 306.6 mg/g (15.3 g/L) and 210 mg/g (10.5 g/L) with untreated, fungal pretreated, alkali pretreated, and acid pretreated BS, respectively, in 48 h. Chapla et al. (2010) reported alkali (dilute NaOH) pretreated WS and rice straw yielded 151.6 and 163.06 mg/g of sugar, respectively. Taniguchi et al. (2005) reported the release of 330 mg/g of sugar from rice straw fermented with *Pleurotus ostreatus*. Moreover, Gupta et al. (2011) have reported release of 402 mg/g sugar from *Prosopis Juliflora* fermented with *Pycnoporus cinnabarinus*. Monsalve et al. (2006) reported 20 g/L reducing sugar yield after sequential alkali–acid pretreatment of BS.

The results thus obtained clearly indicate that the pretreatment of substrates resulted in an improved enzymatic saccharification compared to untreated substrates. This may be attributed to the increase in available surface area and decrease in degree of polymerization along with the separation of lignin–carbohydrate linkage for easy accessibility of substrates for enzymatic hydrolysis (Sun and Cheng 2002).

### Ethanol production

The sugar hydrolysate obtained from saccharification of untreated, pretreated WS and BS was investigated for yeast cell biomass and ethanol production (Table 3). Several evidences have suggested that the separate fermentation by substrate-specific organisms worked better instead of using single culture or co-culture (Cheng et al. 2008). The cellulosic hydrolysates were fermented with *S. cerevisiae* NCIM 3570 and the highest ethanol production of 3.38 g/L with a yield of 0.54 g/g was obtained with fungal pretreated WS hydrolysate along with 0.91 g/L of yeast cell biomass. Moreover, fermentation of fungal pretreated BS hydrolysate gave 2.0 g/L of ethanol with yield of 0.40 g/g, after 48 h along with 0.85 g/L of yeast cell biomass. Lower ethanol yield was obtained with untreated WS (0.31 g/g) and BS (0.15 g/g) in comparison with that of the treated one. In the present experiment, the ethanol yield (0.40 g/g) obtained from fungal pretreated BS hydrolysate was very much in agreement with the report of Mamma et al. (1995) who reported 0.40 g/g of ethanol yield from sweet sorghum. However, the ethanol yield (0.54 g/g) from fungal pretreated WS hydrolysate was higher than that reported by having an ethanol yield of 0.48 g/g from corncob (Chen et al. 2007). Reddy et al. (2010) reported 3.82 g/L of ethanol from alkali pretreated BS. The above results, thus show that the pretreatment increases ethanol yield.

**Table 3:** Ethanol production from enzymatic hydrolysates of WS (untreated and pretreated) and BS (untreated and pretreated) by *Saccharomyces cerevisiae* (NCIM 3570)

Substrates	Ethanol production (g/L)	Ethanol yield (g/g)	Yeast cell biomass (g/L)
Untreated (control) wheat straw	1.0 ± 0.03	0.31 ± 0.02	0.53 ± 0.02
Fungal pretreated wheat straw	3.38 ± 0.05	0.54 ± 0.01	0.91 ± 0.05
Alkali pretreated wheat straw	1.95 ± 0.04	0.35 ± 0.02	0.67 ± 0.05
Acid pretreated wheat straw	2.75 ± 0.05	0.40 ± 0.02	0.86 ± 0.04
Untreated banana stem	0.7 ± 0.01	0.15 ± 0.01	0.45 ± 0.01
Fungal pretreated banana stem	2.0 ± 0.03	0.40 ± 0.03	0.85 ± 0.03
Alkali pretreated banana stem	3.8 ± 0.05	0.35 ± 0.02	1.23 ± 0.05
Acid pretreated banana stem	1.9 ± 0.03	0.20 ± 0.01	0.59 ± 0.01

## Conclusion

The present study demonstrates that the pretreatment methods are a remarkable approach for massive utilization of lignocellulosic biomass for higher sugar yield during saccharification and eventually to bioethanol production. The results obtained with the biological pretreatment were better in case of WS and BS with respect to the yield of ethanol which shows the potentiality of our culture *Pleurotus ostreatus* HP-1 which posses a good ligninolytic and lignin degrading ability which lead to the significant improvement of enzymatic hydrolysis of WS and BS. Thus, the utilization of lignocellulosic biomass for bioethanol production necessitates the production technology to be cost effective and environmentally sustainable. Process integration (process engineering and strain engineering) for second generation bioethanol will still need to be carried out to circumvent the difficulties of co-fermentation (pentose and hexose) and thus, to improve system efficiency.
